# Deciphering the Role of microRNA Mediated Regulation of Coronin 1C in Glioblastoma Development and Metastasis

**DOI:** 10.3390/ncrna9010004

**Published:** 2023-01-04

**Authors:** Denis Mustafov, Emmanouil Karteris, Maria Braoudaki

**Affiliations:** 1 School of Life and Medical Sciences, University of Hertfordshire, Hatfield AL10 9AB, UK; 2 College of Health, Medicine and Life Sciences, Brunel University London, Uxbridge UB8 3PH, UK

**Keywords:** CORO1C, mRNA, microRNA expression, brain, cancer, glioblastoma, pediatric, adult

## Abstract

Glioblastoma multiforme (GBM) is a highly heterogenic and malignant brain tumour with a median survival of 15 months. The initial identification of primary glioblastomas is often challenging. Coronin 1C (CORO1C) is a key player in actin rearrangement and cofilin dynamics, as well as enhancing the processes of neurite overgrowth and migration of brain tumour cells. Different bioinformatic databases were accessed to measure CORO1C expression at the mRNA and protein level in normal and malignant brains. CORO1C expression was observed in brain regions which have retained high synaptic plasticity and myelination properties. CORO1C was also expressed mainly within the hippocampus formation, including the Cornu Ammonis (CA) fields: CA1–CA4. Higher expression was also noticed in paediatric GBM in comparison to their adult counterparts. Pediatric cell populations were observed to have an increased log2 expression of CORO1C. Furthermore, 62 miRNAs were found to target the CORO1C gene. Of these, hsa-miR-34a-5p, hsa-miR-512-3p, hsa-miR-136-5p, hsa-miR-206, hsa-miR-128-3p, and hsa-miR-21-5p have shown to act as tumour suppressors or oncomiRs in different neoplasms, including GBM. The elevated expression of CORO1C in high grade metastatic brain malignancies, including GBM, suggests that this protein could have a clinical utility as a biomarker linked to an unfavorable outcome.

## 1. Introduction

### 1.1. Epidemiology and Molecular Characteristics

Presented with a 5-year survival rate of 5.6%, glioblastoma multiforme (GBM) remains one of the most aggressive and deadliest glial malignancies amongst adults [1]. The median onset of GBM is 64 years; however, the malignancy can occur at any age indiscriminatory [2]. GBM accounts for nearly 3350 newly diagnosed cases in the UK annually with an overall survival rate between 6–17 months. Proclaimed as an incurable brain tumour due to its infiltrative nature, complete surgical resection of the tumour burden is almost impossible, and thus inevitable progression of residual infiltrates enhance relapse and reoccurrence of the malignancy [3]. Postoperative radiotherapy and adjuvant chemotherapy with temozolomide are subsequently prescribed post tumour resection.

Paediatric glioblastomas (pGBM) possess distinctive molecular characteristics, which distinguish them from their adult counterparts. pGBM is a rare event accounting for 3–7% of all primary central nervous system (CNS) tumours in children [4]. The estimated median overall survival rate for paediatric GBM is 10.5 months, with children over 10 years of age being impacted more, due to a higher frequency of mutations detected when compared to younger children. A previous study on pGBM revealed that younger children had better overall survival outcomes [5]. Despite the fact that there is limited knowledge about the tumour microenvironment of paediatric GBM, the previous data demonstrated that this cohort of patients presented with significant alternations in mismatch repair (*MMR*) genes, such as *MSH2* and *MSH6* [6]. Contradictive evidence regarding the isocitrate dehydrogenase 1 (*IDH1*) status in primary paediatric glioblastomas suggested that almost all cases within young children were *IDH*-wild type, whereas an adolescent’s cohort with secondary paediatric GBM presented with an *IDH*-mutant molecular profile [7,8]. Despite the rarity of the malignancy, an exceptional study evaluating a cohort of 1173 pGBM could be subcategorised into several pathological entities based on the age of occurrence, diagnostics, molecular alternations, and location. These include paediatric high-grade glioma (PHGG), diffuse intrinsic pontine glioma (DIPG), and pediatric low-grade glioma (PLGG), with the latest being the most common type of CNS tumour found in children [9]. Gross resections of HGG have shown improved survival rates within patients, whereas such are unfeasible for DIPG due to the location of the tumour [10]. A further difference associated with the various survival rates within the pGBM population is the presence of *H3F3A* at the K27 mutation occurring in adolescents. This mutation defines a new subtype, diffuse midline glioma, which possesses distinct clinical and epigenetic profiles [11].

The undefined therapeutic approach for pGBM patients in comparison to their adult GBM counterparts has led to indistinguishable therapy routines, despite the inherent differences in their molecular profiles. However, radiation is often deferred for children’s therapy, especially in infants, due to concerns for potential damage to the developing brain [12]. The location of pGBM also differs, with most tumours developing within the thalamus and brainstem leaving surgeons with limited routes for safe surgical resection [13].

### 1.2. MicroRNAs and Their Role in Glioblastoma Tumorigenesis and Diagnosis

MicroRNAs (miRNAs) are described as highly conserved, short, non-coding fragments of RNA (ncRNA) which constitute of around 22 nucleotides in length. Discovered nearly three decades ago, miRNAs are now recognised as post-transcriptional regulators for just over 60% of all protein-coding genes [14]. The biogenesis of miRNAs begins with post- or co-transcriptional processing of RNAse polymerase II/III [15]. Most intragenic miRNAs are transcribed from the introns of protein-coding genes, whereas very few are synthesised from the exons of such genes. Those miRNAs found to be intergenic have their own promoter regions and are transcribed autonomously to their host genes [16].

Validating miRNA biomarkers might provide a major advancement in the identification and treatment for brain cancer patients, with the hope they could predict how each patient’s tumour is going to behave and respond to a particular targeted treatment. However, their clinical adaptation is still far from accepted, due to uncertainties in the experimental findings caused by the limited number of patient samples used in research [17]. Some miRNAs can act as tumour suppressors and others as oncogenes (oncomiRs). Detection of miRNA signatures in primary brain tumours has revealed unique avenues for assessing the diagnosis, prognosis, and monitoring of patients [18]. A systematic review has revealed that more than 300 miRNAs are affected in GBM tumorigenesis, with 253 being overexpressed and 95 under-expressed [19]. The short size of miRNAs allows them to bind imperfectly or complimentary to more than one mRNA and subsequently to regulate more than one target. It has been estimated that approximately 10,000 mRNAs can be regulated via this mechanism, allowing a single miRNA to be a direct or indirect target for multiple genes [20]. The deregulation of miRNAs in cancer is aided by different abnormalities in the biogenesis of miRNAs, such as amplification, deletion, epigenetic modification and silencing of miRNAs, translocation, and defective biosynthetic enzymes, that lead to either overexpression or downregulation.

Despite efforts made to combat deadly glioblastoma multiforme tumours, these types of brain cancers are still associated with poor prognosis and low overall survival rates. MiRNAs have demonstrated promising outcomes in their use as diagnostic, prognostic, and treatment agents. Elucidating aberrant miRNA expression in brain malignancies might provide novel biomarkers related to diagnosis, prognosis, and tailored therapy. For instance, miR-21 has been found to be overexpressed in many types of cancer, such as GBM, colorectal, and breast cancer. Nevertheless, miR-21 plays a pivotal role in the tumorigenesis of GBM and is the only miRNA which has clearly defined diagnostic and prognostic properties to date. The levels of miR-21 drastically decrease post tumour resection, allowing the biomolecule to be used as a monitoring agent for patients and to detect early relapse [21]. Other miRNAs with potential diagnostic properties include miR-128 and miR-342-3p [22]. Previous research has confirmed the high analytical specificity and sensitivity of both, miR-21 and miR-128, in their use as diagnostic markers for GBM. For instance, Roth et al. (2011) demonstrated the significant downregulation of miR-128 in the peripheral blood samples of 20 GBM patients with an accuracy of 81%, and sensitivity and specificity of 79% and 81%, respectively [23].

### 1.3. Nanosensor-Based Diagnostic Approaches for GBM

Several approaches are currently incorporated in the diagnosis of GBM. Of these, magnetic resonance imaging (MRI) and computed tomography (CT) are of main use. A known drawback for these methods is that small tumour portions and infiltrations are hard to be detected [24]. Nanosensor-based diagnostic approaches offer a tailored way for the detection of GBM. For instance, molecular MRI utilises multifunctional iron oxide nanoparticles (IONP) which act as imaging agents for GBM and can be directly detected by MRI imaging [24]. These nanoparticles can also act as carriers of tumour-specific biomolecules. Polyethylene glycol (PEG) coated nanoparticles showed increased specificity of MRI contrast enhancement within GBM patients via the epidermal growth factor receptor variant III (EGFRvIII) antibody–IONP complex in vitro [25]. Even though nanosensor-based diagnostic approaches have shown promising results, the appearance of neurotoxicity from the application of nanoparticles remains a major concern. Thus, a careful consideration of unwanted side effects and the removal of nanoparticles from the brain should be comprehensively evaluated before their clinical utilisation.

### 1.4. Coronin 1C and Its Role in Glioblastoma Multiforme

Coronin 1C (CORO1C) has been shown to play a key role during the processes of migration and proliferation via its action upon actin rearrangement and cofilin dynamics. Potential miRNA tumour suppressors might aid helpfully in targeting *CORO1C*, rescuing its oncogenic phenotype. Previous research has identified that CORO1C is expressed significantly within migrating cells and its silencing demonstrated reduced proliferation and migration velocity within the glioblastoma cell lines A172 and U373 [26]. However, there is limited information on how CORO1C regulates these processes within glioblastoma patients. *CORO1C* was also found to be a direct target of miR-128a, showing upregulation when miR-128a was silenced [27]. However, the regulation of *CORO1C* via miRNAs is not fully examined and possible avenues for identifying novel miRNA targets exists.

The aforementioned existing differences between pediatric and adult glioblastomas’ molecular profiles require new approaches for the diagnosis and treatment of the malignancy in different patient cohorts. Identifying a common, differentially expressed molecular biomarker for both pGBM and adult GBM could possibly aid in the early detection of GBM and provide a common therapeutic target.

In this in silico study, we sought to investigate the differential expression of the CORO1C protein across the brain profiles of paediatric and adult GBM patients. We demonstrated that the CORO1C mRNA expression was elevated within specific brain regions and within paediatric GBM patients, associated with highly aggressive brain neoplasms, suggesting that it potentially possesses oncogenic properties.

## 2. Results

### 2.1. CORO1C Normal Tissue Distribution

**Figure 1:**
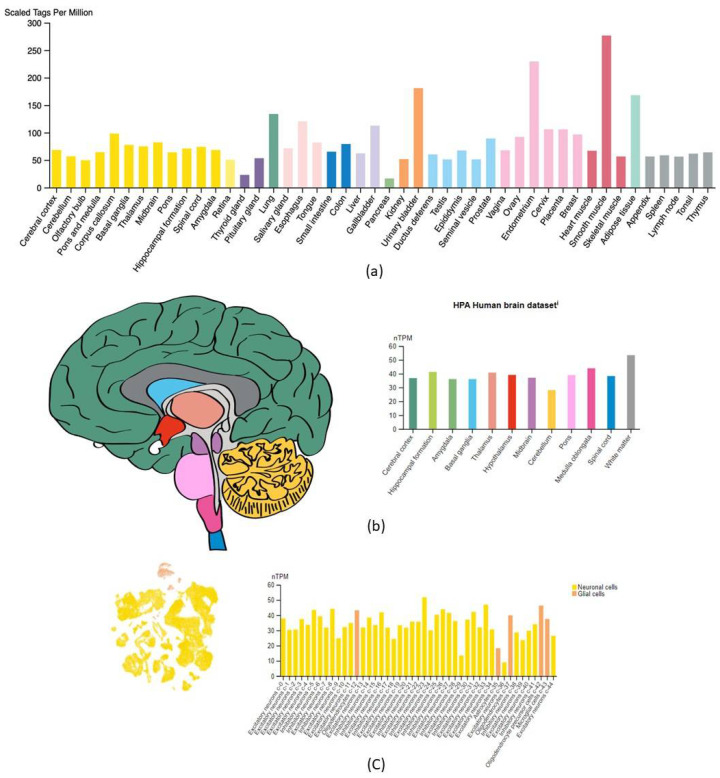
Detection of *CORO1C* mRNA across normal tissue types and brain [28,29]. (**a**) The Human Protein Atlas database was incorporated to examine the detection of *CORO1C* mRNA expression within different tissue types. Higher expression was observed within smooth muscle tissue, endometrium, urinary bladder, adipose, and lung tissues. (**b**) The average distribution of *CORO1C* mRNA throughout the brain demonstrated that the protein was highly expressed in the white matter, medulla oblongata, and hippocampal formation regions of the brain. (**c**) Single-cell cluster analysis obtained from normal brain tissue demonstrated high expression of *CORO1C* amongst neuronal cells, oligodendrocytes, oligodendrocyte precursor cells, and microglial cells.

Figure 1 represents the mRNA expression profile of *CORO1C* within normal tissues. Specifically, in Figure 1a, the highest expression measured in normalized transcripts per million (nTPM) was detected in smooth muscle tissue (276.6 nTPM), followed by the endometrium (229.5 nTPM), urinary bladder (180.8 nTPM), adipose tissue (168.1 nTPM), and lung (133.8 nTPM). When brain tissue and its associated regions were assessed separately, an average distribution of *CORO1C* of 39.2 nTPM across all regions was noted (Figure 1b). High expression of *CORO1C* mRNA was observed within the white matter (53.4 nTPM), followed by the medulla oblongata and hippocampal formation (43.9 and 41.3 nTMP, respectively). Single-cell analysis of the brain illustrated that *CORO1C* was highly expressed within neuronal cells. The glial cluster comprising of astrocytes, oligodendrocytes, oligodendrocyte precursor cells, and microglial cells revealed that CORO1C was highly expressed within all types of glial cells except for astrocytes (Figure 1c).

### 2.2. CORO1C Regional Localization

**Figure 2:**
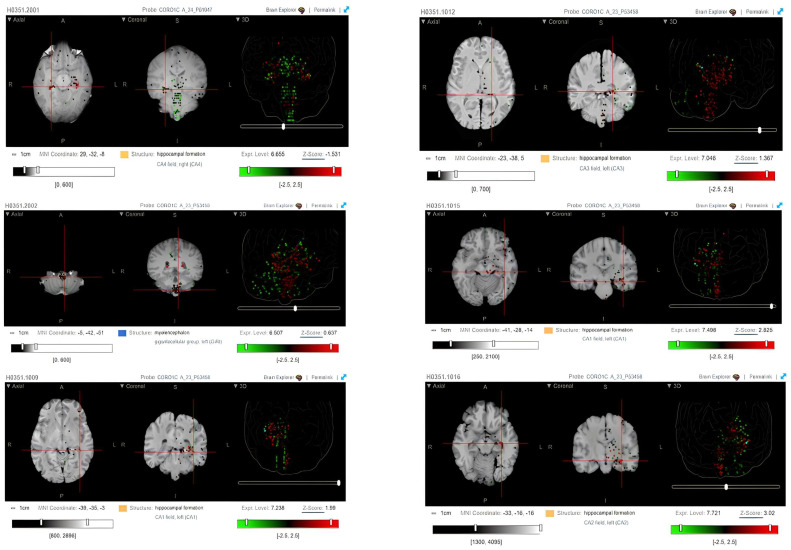
Heatmaps of log2 expression values of six patients obtained from microarrays experiments (Allen brain atlas). The Allen brain atlas database revealed that CORO1C is highly expressed within the hippocampal formation and myelencephalon regions of the brain [30].

The expression of CORO1C within the different brain regions of six patients was also evaluated via heatmaps of log2 expression values obtained using microarrays. High ex-pression of CORO1C is noticed within the hippocampal formation region of the brain. Figure 2 represented the Magnetic Resonance Imaging (MRI) scans and heatmaps from six healthy individuals who have succumbed from causes other than brain tumors. Ex-pression was mainly present across the CA1 (log2 expression 7.2 ± 2.5 and 7.5 ± 2.5), CA2 (log2 expression 7.7 ± 2.5), CA3 (log2 expression 7.0 ± 2.5), and CA4 (log2 expression 6.7 ± 2.5) fields, which are parts of the hippocampus. As observed in the heatmaps, high expression levels of CORO1C were also observed within the myelencephalon region of the brain (log2 expression 6.5 ± 2.5).

### 2.3. CORO1C within Various Cancer Types

**Figure 3:**
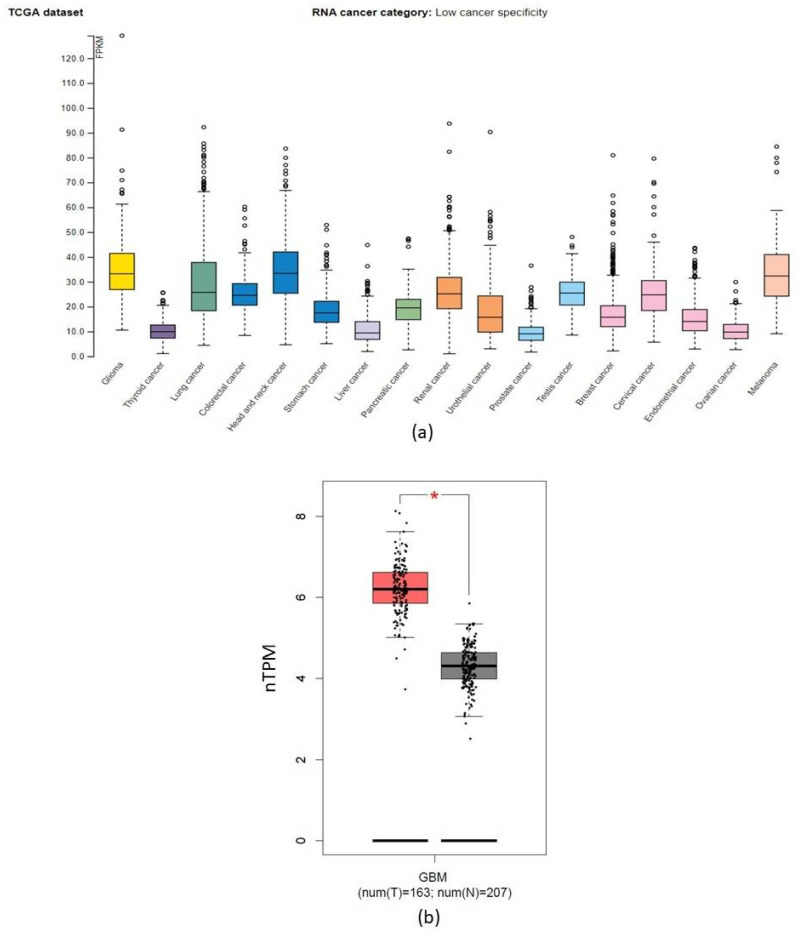
Expression of *CORO1C* in various cancerous tissues. (**a**) *CORO1C* expression across 17 cancer types revealed that the gene was highly expressed in head and neck cancer, gliomas, and melanomas. ( **b**) *CORO1C* was significantly upregulated in GBM tumour samples (red) in comparison to normal brain tissue (grey) (One way ANOVA, * *p* < 0.01).

Figure 3 represented RNA-seq data available to the TCGA database to identify the degree of specificity of CORO1C in diverse cancer types. The RNA-seq data was expressed in a median number of fragments per kilobase of exon per million reads (FPKMs). Amongst the 17 different cancer types, the FPKM median expression was highest in head and neck cancers and gliomas, 33.4 FPKMs and 33.2 FPKMs, respectively. CORO1C expression determined by RNA-seq was also high in melanoma cancer, 32.3 FPKMs. Further analysis of CORO1C expression within normal and cancerous brain samples confirmed that CORO1C was significantly overexpressed within the brain tumour cohort, *p* < 0.01 (Figure 3b).

### 2.4. CORO1C Comparative Analysis of Expression between Paediatric and Adult GBM Cohorts

**Figure 4:**
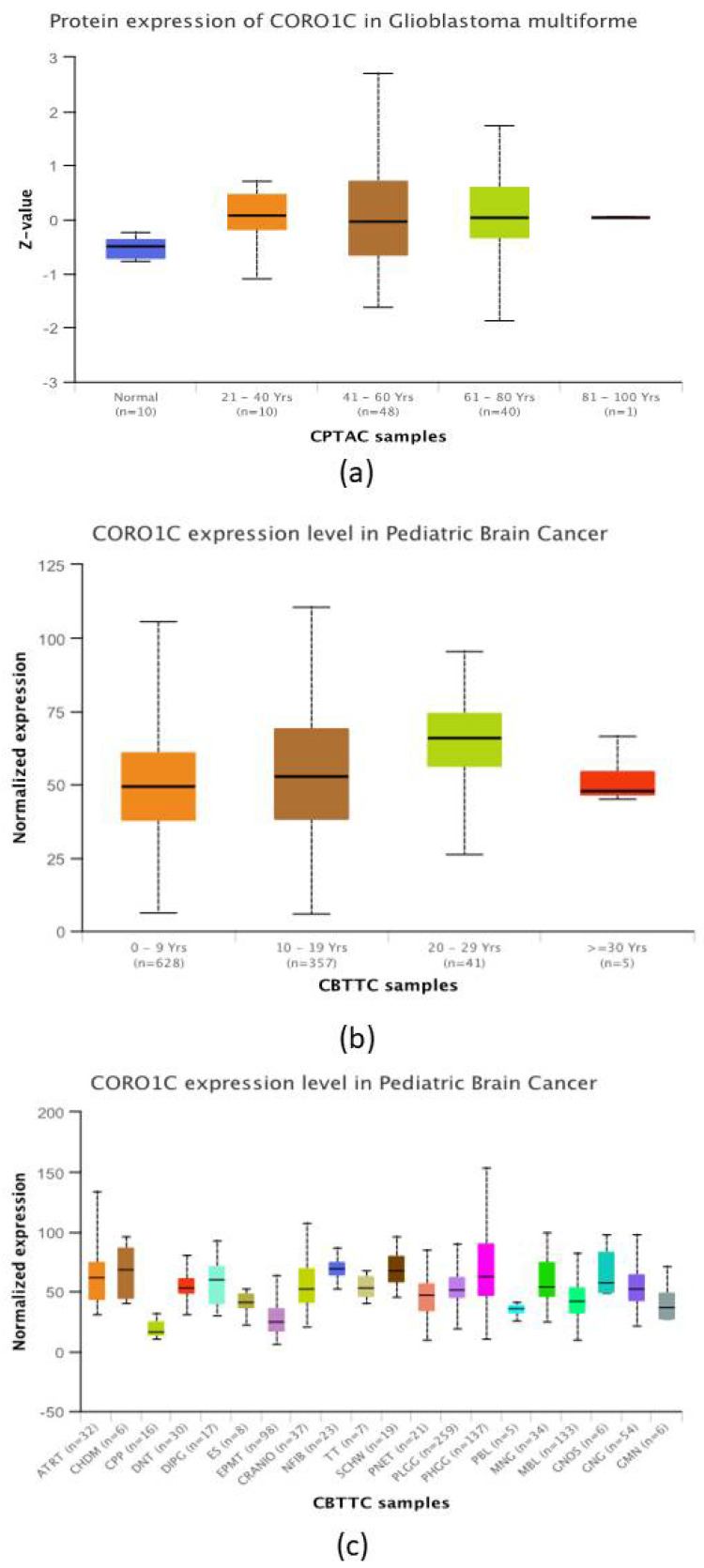
UALCAN comparative analysis of the expression of CORO1C within adult and paediatric glioblastoma patients. (**a**) Significantly high total protein levels of CORO1C were observed in all age groups compared to a normalised sample, except age group 81–100 years (normal versus age 21–40 years, *p* < 6.015973 × 10^−3^; normal versus age 41–60 years, *p* < 4.724525 × 10^−4^; normal versus age 61–80 years, *p* < 1.753039 × 10^−3^). (**b**) CORO1C expression was higher in age groups 0–9 years, 10–19 years, and 20–29 years in comparison to their counterparts at age ≥ 30 years of age. However, there was no statistical significance of expression between the different age groups. (**c**) Neurofibroma, chondroma, schwannoma, PHGG, and DIPG showed highest expression levels of CORO1C. Neurofibromas had significantly higher expression of CORO1C in comparison to PHGG (*p* < 3.217085 × 10^−2^). PLGG, DIPG, CRANIO, PTEN, ES, DNT, and CPP subtypes have significantly lower expressions of CORO1C in comparison to PHGG (PLGG versus PHGG, *p* < 2.50863678369934 × 10^−7^; DIPG versus PHGG *p* < 3.535811 × 10^−3^; CRANIO versus PHGG *p* < 5.516405 × 10^−3^; PTEN versus PHGG *p* < 2.994696 × 10^−3^; ES versus PHGG *p* < 5.98540311685281 × 10^−8^; DNT versus PHGG *p* < 1.163218 × 10^−2^; CPP versus PHGG *p* < 1.75021275936207 × 10^−18^).

The expression of CORO1C based on patients’ ages is illustrated in Figure 4a. Three GBM age groups ranging from 21–80 years of age had significantly increased CORO1C total protein expression levels when compared to normal patient samples. The highest significance was observed between normal samples versus the GBM age group 41–60 years, followed by the age groups 21–40 years and 61–80 years, respectively. Panel 4B provided a deeper insight on the expression levels of CORO1C within paediatric GBM samples. The age groups of 0–9 years, 10–19 years, and 20–19 years had higher median expression of CORO1C when compared to their counterparts at age ≥ 30 years (49.3 nTPM, 52.6 nTPM, 66.0 nTPM in comparison to 47.9 nTPM, respectively). However, no significant difference was observed. The expression of CORO1C in different types of paediatric brain malignancies is shown in Figure 4c. The highest median expression of the protein was detected in neurofibroma, chondroma, schwannoma, PHGG, and DIPG (70 nTPM, 69.4 nTPM, 68.0 nTPM, 62.8 nTPM, and 60.7 nTPM, respectively). This reached significance in neurofibromas only, when compared to PHGG. The overall expression levels of CORO1C within PLGG, DIPG, CRANIO, PTEN, ES, DNT, and CPP paediatric brain cancer subtypes were significantly lower in comparison to the expression of the protein found in HPGG.

### 2.5. CORO1C Single-Cell Expression Analysis in Various GBM Cell Clusters

**Figure 5:**
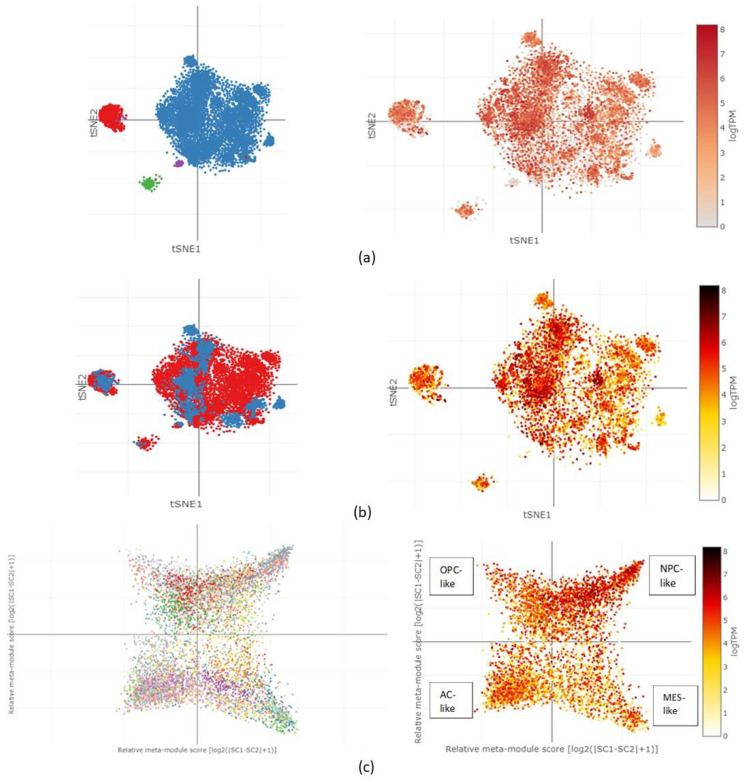
Single-cell analysis of glioblastoma cell clusters from paediatric and adult patients. (**a**) tSNE heatmap showed CORO1C expression within the left half of the malignant cell cluster (blue), almost exclusively across the macrophage cluster (red) and centre of the oligodendrocyte cell cluster (green). Little or no expression was observed within the T-cell cluster (purple). (**b**) Expression of CORO1C within the paediatric cluster (blue) was higher, ranging between log2 of 6 and log2 of 8, when compared to cells from the adult cluster (red). (**c**) Expression of CORO1C within astrocyte like (AC-like), oligodendrocyte precursor cells (OPC-like), mesenchymal like (MES-like), and neural-progenitor-like cells (NPC-like) demonstrated global expression across all cellular states with NPC-like state showing slightly higher expression of the protein (>log2 of 6).

The different types of cell clusters obtained from 28 individuals spanning both adult and paediatric GBM cohorts included macrophages, T-cells, malignant cells, and oligodendrocytes. In Figure 5a, the tSNE heatmap showed CORO1C expression within the left half of the malignant cell cluster, almost exclusively across the macrophage cluster and centre of the oligodendrocyte cell cluster. Limited expression was observed in the T-cell cluster. The expression of the protein was between log2 of 4 and log2 of 8 in these areas. A comparison between 8 paediatric cell populations and their 20 adult counterpart cell populations was performed as shown in Figure 5b. Expression of CORO1C within the paediatric cluster was higher, ranging between log2 of 6 and log2 of 8, when compared to cells from the adult cluster. The results revealed that paediatric cells occupied the left side of the malignant cell cluster, almost entirely the macrophage cluster and the centre of the oligodendrocyte cell cluster, an observation stated above regarding the increased log2 expression of CORO1C within these regions of the clusters. The expression of CORO1C was evaluated via the two-dimensional representation of each cellular state, including astrocyte like (AC-like), oligodendrocyte precursor cells (OPC-like), mesenchymal like (MES-like), and neural-progenitor-like cells (NPC-like). The heatmap of the log2 expression in Figure 5c revealed that CORO1C was expressed globally across all cellular states with an expression level of more than 4 logTPM. The expression of the protein within the NPC-like state was slightly higher in comparison to the rest of the cellular states: more than 6 logTPM, respectively.

### 2.6. miRNA Targets and Physical Interactions of CORO1C

**Figure 6:**
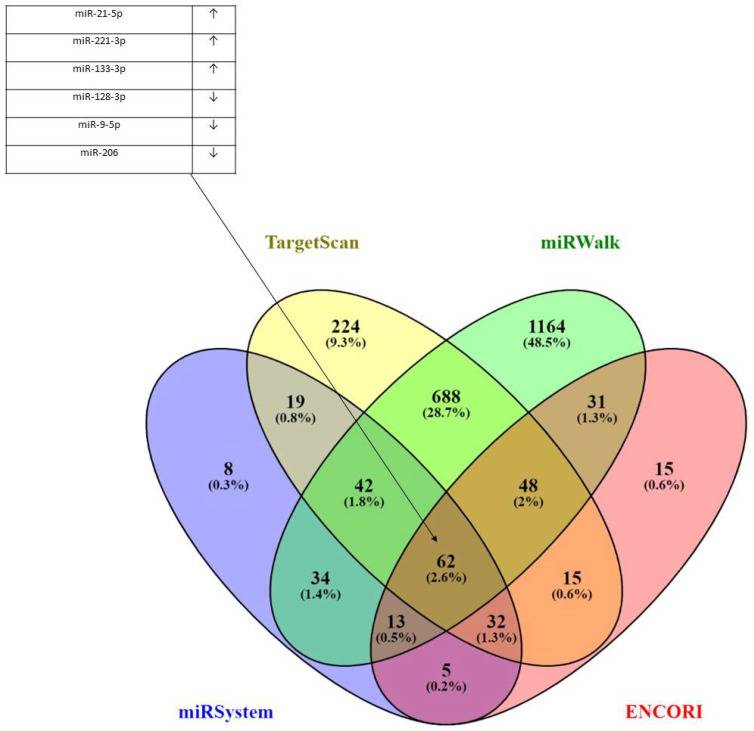
miRNA gene targets for *CORO1C* and physical interactions of *CORO1C* and other genes. Venn diagram incorporating miRSystem, TargetScan, miRWalk, and ENCORI databases. In total, 62 miRNAs were found to overlap between the 4 miRNA analysis tools (2.6%). Of these, miR-133-3p, hsa-miR-206, and miR-128-3p have been shown to be associated with *CORO1C* gene expression in different types of neoplasms.

Four databases were incorporated in finding common target miRNAs for the CORO1C gene, including miRSystem, TargetScan, miRWalk, and ENCORI. The results presented in the form of a Venn diagram shown in Figure 6 demonstrated that there were 62 (2.6%) common miRNA targets when all four miRNA analysis tools were used. Some of these common miRNAs included downregulation of hsa-miR-34a-5p, hsa-miR-128-3p, miR-9p, and miR-181a/b/d-5p and upregulation of hsa-miR-221-3p, hsa-miR-125p, and hsa-miR-21-5p and sha-miR-141-3p in glioblastomas. All six miRNAs have been previously shown to play key functions in glioblastomas. Also, miR-133a-3p and miR-206 were found overexpressed in the majority of the cancer types screened. The full list of all 62 miRNAs can be found in Supplementary Table S1.

## 3. Discussion

The oncogenic properties of CORO1C have been described in several types of cancer, including colorectal and liver cancers, non-small cell lung carcinoma, and melanomas [31,32,33,34]. The regulation of actin dynamics is essential for some disease states, including migration, cell polarity, signal transduction, and intracellular trafficking. Coronins are a family of highly conserved proteins that play an important role in cell motility and vesicle trafficking. *CORO1C* is a serum-induced gene and external stimuli, such as serum and growth factors, could up-regulate the transcriptional activation of the gene. However, little is known about the exact molecular mechanism that leads to increased transcriptional events. The gene product has emerged as a key player in actin rearrangement and cofilin dynamics via its effects upon the assembly of actin-related proteins 2/3 (Arp2/3). Previous evidence has revealed that CORO1C exerts its effects via F-actin turnover during the processes of neurite overgrowth and migration of brain tumour cells [35]. The expression of *CORO1C* in astroglia cells and tumour cells is related to migrative and proliferative phenotypes. High levels of the protein are also associated with an increased grade of the malignancy, whereas neurons in the cortex of the brain show no significant expression of *CORO1C* [36]. The aim of this study was to in silico analyse the expression profiles of CORO1C in glioblastomas and to identify its diagnostic or prognostic potential.

Our results demonstrate that the average distribution of CORO1C throughout the brain was highest within the white matter, medulla oblongata, and hippocampal formation regions of the brain. CORO1C was also expressed amongst neuronal cells, oligodendrocytes, oligodendrocyte precursor cells, and microglial cells. Our results regarding the regional distribution of CORO1C, assessed by the Allen brain atlas also suggested overexpression of CORO1C within normal brain tissue, was present mainly in the hip-pocampal formation region of the brain, including the CA1-CA4 fields. Hippocampal pyramidal neurons and Purkinjean cells were shown to retain high levels of CORO1C during murine brain development. This feature enhanced a high level of synaptic plasticity which is inevitably linked to the processes of learning and memory [27]. CORO1C expression is also linked to the process of myelination, hence its high mRNA expression observed within the white matter, followed by the medulla oblongata and hippocampal formation. The localised expression of CORO1C suggested that the protein played an important role in differentiation, migration, the formation of neurites, and myeline sheets in oligodendrocytes [37]. Hasse et al. (2005) carried out in vitro experiments with neuro-2a and PC-12 cells transfected with GFP-tagged CORO1C versions. The team concluded that full-length CORO1C localised and supported outgrowing neurites, whereas truncated forms of the protein acted as suppressors for neurite formation. Thus, CORO1C appears to play a major role in the morphogenesis of neuronal cells [38]. The primary location of pGBM formation, the supratentorial brain, is found in close proximity to the CA fields. This is in line with our findings suggesting an elevated expression of CORO1C within areas near to the supratentorial region. On the other hand, adult GBM neoplasms are commonly found in cerebral hemispheres, especially in the frontal and temporal lobes of the brain. Our results did not show differential expression of CORO1C within these regions, suggesting that CORO1C levels might be elevated in pGBM cases due to their regional distribution. These findings might serve as a guidance for the clinical diagnosis of pGBM cases.

In the current study, overexpression of CORO1C in both adult and pediatric glioblastomas was observed. This is in line with Thal et al. (2008), who demonstrated high ex-pression of CORO1C in diffuse gliomas. They specifically showed that in normal brain tissue, the expression of CORO1C in cortex neurons was significantly reduced, whereas high grade benign brain neoplasms expressed increased levels of the protein. Thus, the expression of CORO1C is possibly dependent on tumour type and grade [36]. An experimental setting comprised from both in vitro and in vivo studies, demonstrated that CORO1C could promote glioma growth via the Wnt/β-catenin signalling pathway [39]. The overexpression of CORO1C in aggressive solid tumours such as glioblastomas and head and neck cancers are often associated with its metastatic powers [33]. In associations between 17 cancer types, the “crown-like” protein has exhibited the highest expression in head and neck cancers and gliomas. Nevertheless, it is not yet exclusively clear how CORO1C could enhance the metastatic and proliferative properties of GBM. Similar expression profiles have been observed in other types of malignancies. For instance, high expression of CORO1C has been observed in melanomas when compared to normal skin tissue. Specifically, evidence demonstrated that increased levels of the mRNA were found in BRAF mutant melanomas with high phosphor-Erk levels [31]. The frequent up- or down-regulation of CORO1C observed in microarray experiments of different cancer types, such as in glioblastomas and colorectal cancer (CRC) suggested that the protein is highly transcriptionally dynamic. As a member of a conserved protein family, the actin-binding CORO1C has been shown to interact with trophoblast cell surface protein 2 (Trop2) in colorectal cancer [32]. Trop2 is an oncogenic protein that plays a vital role in stimulating the aggressive behavior of CRC. In the same research, it was also suggested that the overexpression of CORO1C in CRC patients was associated with lymph metastasis, distant metastasis, venous invasion, and perineuronal invasion. These findings suggested that elevated CORO1C might act as a prognostic factor for early metastasis events in different types of neoplasms and its clinical utilization as such could be beneficial for the early detection of metastatic formations.

The results obtained from UALCAN revealed that CORO1C was significantly overexpressed within patient groups in comparison to their normal counterparts, apart from the 81–100 years age group samples. However, a limitation of the study is that this age group is comprised from a limited number of patients. A comparison of the expression level of CORO1C within paediatric and adult GBM patients was also performed and differences were observed between younger ages and the > 30 years of age group; however, these did not reach significance. Previously, large molecular profiling studies have pointed out distinct molecular landscape differences between paediatric and adult glioblastomas [40,41]. Key driver epigenetic mutations within isocitrate dehydrogenase (*IDH1*) are classed as causative in adult GBM, and alterations in the *TP53*, *TERT*, and *ATRX* genes act as common prognostic and therapeutic targets. Unlike their adult counterparts, paediatric patients with GBM are likely to present with cancer predisposition syndromes, such as mismatch repair deficiency syndrome (CMMRD) [42]. A variety of malignant neoplasms are characteristic for children with CMMRD, including brain, gastrointestinal tract, and haematologic malignancies with a median onset of 7.5 years of age [43]. The difference between paediatric and adult GBM levels of CORO1C expression might be caused due to the high degree of neuronal plasticity found in children’s developing brains. Another factor might be the high tumour mutational burden, which is characteristic for children with CMMRD. In most cases, such paediatric patients benefit from immune therapies, which commiserates the urgent need of early diagnostic and prognostic biomarkers.

A CBTTC dataset of the expression of CORO1C in different paediatric brain tumours depending on their histologic subtype was also incorporated in this research. Based on our results, the significantly highest median expression of the protein was obtained in the neurofibroma when compared to PHGG. Neurofibromas are benign nerve sheath brain tumours that grow on nerve cells and form soft bums under the skin [38]. Nevertheless, this type of benign malignancy has the potential to metastasize across the head and neck, and skin regions [44]. As aforementioned, CORO1C levels remain high in regions of myelination and differentiation. This finding is in agreement with the analysis using The Cancer Atlas dataset, which also revealed that the expression of CORO1C was highest in head and neck cancers, followed by gliomas and melanomas. The presence of CORO1C amongst these neoplasms might aid a possible poor prognostic biomarker associated with the metastatic capacity of the tumour and its spread. Other paediatric histological neoplasms, such as LGG, craniopharyngiomas, dysembryoplastic neuroepithelial tumours (DNT), and choroid plexus papilloma (CPP) are examples of lower grade brain tumours that grow slowly and are benign in most cases. The mRNA expression of CORO1C in these malignancies was significantly lower when compared to the highly malignant HGG neoplasms. This further supported the evidence that CORO1C could be exponentially expressed in tumours with malignant and invasive properties and hence could serve as a biomarker associated with an unfavorable prognosis. Furthermore, the presence of increased CORO1C levels within highly malignant pGBM cases correlates with the fact that the protein is expressed in brain regions with high synaptic plasticity. The factors concerning brain development and plasticity during childhood might serve as a possible explanation why CORO1C levels were found to be elevated in pediatric patients when compared to their adult counterparts. These findings could potentially improve the distinguishment of highly malignant tumors at an earlier stage and improve a patient’s prognosis.

The single-cell RNA-sequencing dataset generated by Neftel et al. (2019) allowed the detection of CORO1C within different cell clusters obtained from individuals spanning both adult and paediatric patient cohorts [45]. CORO1C expression was evaluated amongst the intra-tumoral, heterogenic malignant cells obtained from all patients. Limited expression was noticed in the T-cell cluster. Glioblastoma tumours act as suppressors of the innate and adaptive immune systems [46]. The absence of CORO1C across the T-cell cluster might be explained by the fact that GBM neoplasms suppress T-cells, allowing neoplastic cells an immune-checkpoint escape and further proliferation. As CORO1C is required for the normal migration and proliferation of cells aiding its actin-binding properties and its involvement in the formation of neuronal lamellipodia, the presence of the protein within migrating malignant GBM cells is expected to be increased. Except for their supportive role within the brain, glial cell subtypes, such as microglial cells, are involved in the constant screening of the parenchyma of the brain [47,48]. Their dynamic action requires frequent cytoskeletal rearrangements, aided in part by CORO1C. Thus, the increased expression of CORO1C in brain microglial cells is expected, and observed in our findings. When the filter of the clusters was adjusted to show adult and paediatric cell populations, it became apparent that the paediatric cell population occupies the left side of the malignant cell cluster, almost entirely the macrophage cluster, and the centre of the oligodendrocyte cell cluster, an observation stated above regarding the increased log2 expression of CORO1C within these regions of the clusters. The high expression of CORO1C during brain development demonstrated specific expression patterns that correlated with our findings and supported the higher expression of the protein in paediatric patients when compared to their adult counterparts.

When the expression of CORO1C was evaluated via the two-dimensional representation of each cellular state, including AC-like, OPC-like, MES-like, and NPC-like, the heatmap of log2 expression demonstrated that CORO1C was expressed globally across all cellular states with an expression level of more than 4 logTPM. However, the expression of the protein within the NPC-like state was slightly higher in comparison to the rest of the cellular states. The increased expression of CORO1C in NPC-like cells was supported by the fact that these cells give rise to many, if not all, glial and neuronal cells found across the CNS; thus, they can differentiate. There is evidence that glioblastoma cells influence the migration of NPC-like cells towards the tumour site and enhances the expression of transformed phenotypes [34]. Though, not much is known about the process of communication between NPC-like cells and glioblastoma tumour cells [45].

The role of miRNAs hasn regulating the expression of CORO1C in different malignancies provides a novel way of targeting the deregulated expression of the gene and its protein product. A Venn diagram was generated using common target miRNAs from four miRNA bioinformatic tools. These included downregulation ofhasa-miR-128-3p, miR-9-5p, and miR-133a-3p and upregulation of hsa-miR-221-3p, hsa-miR-206, and hsa-miR-21-5p. All six miRNAs have been previously shown to play key functions in glioblastomas [49,50,51,52,53,54,55].

miR-128 was shown to possess an oncogenic property within breast cancer patients via enhancing proliferation and migration, and inhibiting apoptosis [56]. On the contrary, miR-128 has also been found downregulated in other types of malignancies, including glioblastomas and bladder cancer [57]. Restoring the normal levels of the brain-specific miR-128 within GBM has indicated tumor suppressive effects mediated through an interaction with the E3F3a and BMI1 genes and reducing the levels of proliferation and invasiveness [49]. Previously, CORO1C was found to be a direct target of miR-128a, and the protein product of the gene was subsequently enhanced when miR-128a was downregulated in GBM [58]. To the best of our knowledge, this is the only study that has previously identified an association between miR-128 and CORO1C by screening only a limited number of GBM patients. Overexpression of miR-128 was found to decrease the proliferative properties of NPC-like cells and drive their differentiation into neurons [58]. These findings, confirmed both in vitro and in vivo, suggested that miR-128 is an important player in the developing neocortex. This is in line with our findings, which showed high expression of CORO1C within NPC-like cells obtained from GBM cell clusters. Thus, restoring the miRNA expression profile of miR-128a could potentially cease the activity of the gene product and the invasive potential of GBM cells via its interaction with CORO1C. The processes of cell cycle progression, cell transduction, and migration afforded possible pathways via which the gene might exert its tumorigenic potential. Hence, CORO1C might provide an effective therapeutic approach for GBM patients.

miR-9 has been found to possess dual tumour suppressive and promoting properties in different cancer tissues. For instance, miR-9 might act as an oncomiR in non-small cell lung cancers via its effects upon inhibiting E-cadherin [59]. Low expression of miR-9-5p has been associated with poor prognosis within GBM patient cohorts and thus could serve as a prognostic biomarker [50]. The overexpression of miR-9 in the glioblastoma cell lines U87MG and U251 revealed induction of apoptosis, reduced migration, and invasion within the cells [60]. Furthermore, elevated levels of miR-9-5p were shown to inhibit glioma proliferation by downregulating FOXP2 [61]. An interaction between miR-9-5p and CORO1C has been observed but not demonstrated in osteosarcoma pathogenesis [62]. To the best of our knowledge, currently, there is no evidence available directly linking miR-9-5p and CORO1C in glioblastoma cases. Yet, one can hypothesize that overexpression of miR-9-5p could influence CORO1C and cease its migratory properties.

Low levels of miR-133 were detected within GBM patient samples when compared to matched normal brain tissues [51]. Through in vitro experiments, Xu et al. (2015) demonstrated that the overexpression of miR-133 limited the growth of GBM cells and induced their apoptosis. This is in agreement with previous research indicating a similar role of miR-133a-3p and its interaction with CORO1C in hepatocellular carcinomas [63]. Specifically, it has been identified that overexpression of miR-133a-3p promoted apoptosis in hepatocellular carcinoma cells. Hence, this miRNA probably acted as a tumour suppressor and could potentially serve as a therapeutic target for this type of cancer. The direct interaction of miR-133-3p and CORO1C has not been previously evaluated to the best of our knowledge and this might potentially give rise to further research. On the other hand, there is evidence for the negative regulation of miR-206 in GBM patients upon its direct target Frizzled 7 (FZD7) [52]. Zhou et al. (2019) demonstrated that miR-206 has an inhibitory effect on the Wnt/β-catenin pathway by downregulating FZD7. The role of CORO1C in GBM was directly linked to the β-catenin growth dependent mechanism as part of the Wnt/β-catenin signaling pathway. Thus, restoring the levels of miR-206 to normal might have a potential therapeutic effect by ceasing migration and invasion processes. Demonstrated repressed proliferative and invasive properties of miR-206 within the non-small cell lung carcinoma (NSCLC) cell line A549 suggested that the overexpression of CORO1C was suppressed [64]. Even though this study provided a possible mechanism for a future therapeutic approach in targeting CORO1C in NSCLC, little is known about any induced metastatic properties of CORO1C in this type of cancer.

The aberrantly expressed miR-21-5p has been clearly defined as a diagnostic and prognostic biomarker for GBM. Several studies showed that miR-21 has an up-regulated profile in different types of cancer, including colorectal, prostate, and lung cancers, indicating its role as an oncomiR [65,66,67,68]. A rise in miR-21-5p was observed to increase the migration and invasion potential of glioma cells via increasing SOX2 expression [53]. Lou et al. (2017) showed that when the activity of miR-21-5p was suppressed and when SOX2 was inhibited, the beta-catenin levels decreased, leading to diminished invasiveness and migration. Subsequently, miR-21-5p has a direct effect upon the level of β-catenin. The association of CORO1C and miR-21 has not been well defined; however, acknowledging their involvement in the Wnt/β-catenin pathway initiates a possible avenue for research.

The overexpressed miRNA-221 acts as an oncomiR across diverse cancer types, including glioblastomas, gastric, bladder, and lung cancers [69,70]. Significantly high plasma levels (*p* = 0.0001) of miR-221/222 in glioma patients were associated with the degree of glioblastoma infiltration and a poorer overall survival within 95% of the studied cohort [54]. Possible molecular mechanisms via which the miR-221-3p acted during glioblastoma oncogenesis involve promoting the S-phase of the cell cycle, inhibiting apoptosis, or regulating the invasiveness and proliferation of the cancer. However, the association of miR-221-3p and CORO1C and their occurrence in tandem with GBM has not yet been well defined to the best of our knowledge.

## 4. Materials and Methods

The Human Protein Atlas (HPA) (https://www.proteinatlas.org/ accessed on 30 September 2022) was incorporated to analyse the mRNA expression levels of *CORO1C*. HPA-RNA-seq data was used to demonstrate normal tissue expression of *CORO1C* across different tissue types measured in normalised transcripts per million (nTPM). The tool was also used to generate data of *CORO1C* mRNA expression across 17 different types of cancer. The data was obtained via The Cancer Genome Atlas (TCGA) database and was measured in a number of fragments per kilobase of exon per million (FPKM). A bar chart of a normal neuronal cell cluster was also available at the HPA. mRNA expression of *CORO1C* was measured in nTPM within individual brain cell clusters.

Visualisation of CORO1C expression across six brain donors (NM_014325.2) was performed via using the Allen brain atlas (https://portal.brain-map.org/ accessed on 30 September 2022). The results were presented as heatmaps of log2 expression. The data demonstrated in Figure 2 is publicly available from The Allen brain atlas. The UALCAN database (http://ualcan.path.uab.edu/ accessed on 30 September 2022) was incorporated to assess the different mRNA expressions of CORO1C amongst different GBM age groups and several paediatric brain phenotypes (CBTTC: https://pedcbioportal.kidsfirstdrc.org/study/summary?id=phgg_cbttc accessed on 30 September 2022. The single-cell portal was used to assess the log2 expression of CORO1C within 28 GBM cases (GSE131928), among of which there were 8 from a paediatric cohort and 20 from an adult cohort (https://singlecell.broadinstitute.org/single_cell accessed on 30 September 2022).

Venny2.1.0 was used to generate a Venn diagram of common target miRNAs for CORO1C (https://bioinfogp.cnb.csic.es/tools/venny/ accessed on 15 October 2022). Four different miRNA tools were used to generate miRNAs, which targeted CORO1C. These included miRSystem (http://mirsystem.cgm.ntu.edu.tw/index.php accessed on 15 October 2022), miRWalk (NM_001276471) (http://mirwalk.umm.uni-heidelberg.de/ accessed on 15 October 2022), TargetScan (ENST00000261401.3) (https://www.targetscan.org/vert_80/ accessed on 15 October 2022), and ENCORI (The Encyclopedia of RNA Interactomes, https://starbase.sysu.edu.cn/ accessed on 15 October 2022).

One-way ANOVA statistical analysis was used to compare disease states (Tumour or Normal) by GEPIA. Genes with higher log2FC values and lower q values than pre-set thresholds were statistically significance at *p*-value < 0.05. Significance of the TPM values utilised by UALCAN for the generation of boxplots was determined using a *t*-test PERL script with Comprehensive Perl Archive Network (CPAN) module “Statistics:TTest”. Values of *p* < 0.05 were detonated statistically significant.

## 5. Conclusions

The current in silico investigation demonstrated that the overexpression of CORO1C appeared to be a distinctive feature for solid and highly malignant tumours of different origins, including glioblastomas. A higher expression of CORO1C was found in children with GBM when compared to their adult counterparts. The increased presence of the protein within NPC-like cells suggested high migratory power and potential differentiation of these cells in tumour cells. Expression of CORO1C within phenotypically different pediatric brain malignancies and adult GBM cases demonstrated that this protein could serve as a possible diagnostic biomarker associated with an inferior prognosis within GBM patients. The downregulation of miR-128-3p, miR-9-5p, and miR-133a-3p and upregulation of miR-221-3p, miR-206, and miR-21-5p could afford a potential GBM molecular signature relevant to the diagnosis and treatment to this aggressive type of malignancy.

## Data Availability

All data generated or analyzed during this study are included in this published article.

## Supplementary Materials

The following supporting information can be downloaded at: https://www.mdpi.com/article/10.3390/ncrna9010004/s1, Table S1: Common miRNAs (62) in “miRSystem”, “TargetScan”, “miRWalk”, and “ENCORI”.
