# Supplementary material for: Deciphering the Role of microRNA Mediated Regulation of Coronin 1C in Glioblastoma Development and Metastasis
Source: Noncoding RNA. 2023 Jan 4;9(1):4. doi: 10.3390/ncrna9010004 (PMC9844418; doi:10.3390/ncrna9010004)
Supplement: Supplementary file 1 [file ncrna-09-00004-s001.zip › ncrna-2106945-supplementary.pdf]

Table 1S. Common miRNAs (62) in "miRSystem ", "TargetScan", "miRWalk" and "ENCORI".

hsa-miR-125a-5p
hsa-miR-125b-5p
hsa-miR-128-3p
hsa-miR-129-2-3p
hsa-miR-129-5p
hsa-miR-133b
hsa-miR-135b-5p
hsa-miR-136-5p
hsa-miR-141-3p
hsa-miR-149-5p
hsa-miR-150-5p
hsa-miR-181a-5p
hsa-miR-181b-5p
hsa-miR-181d-5p
hsa-miR-182-5p
hsa-miR-185-5p
hsa-miR-194-5p
hsa-miR-197-3p
hsa-miR-200a-3p
hsa-miR-200b-3p
hsa-miR-200c-3p
hsa-miR-204-5p
hsa-miR-206
hsa-miR-21-5p
hsa-miR-211-5p
hsa-miR-214-3p
hsa-miR-221-3p
hsa-miR-222-3p
hsa-miR-26a-5p
hsa-miR-28-5p
hsa-miR-300
hsa-miR-31-5p
hsa-miR-326
hsa-miR-330-5p
hsa-miR-338-3p
hsa-miR-34a-5p
hsa-miR-34c-5p
hsa-miR-381-3p
hsa-miR-409-3p
hsa-miR-433-3p
hsa-miR-449a
hsa-miR-449b-5p
hsa-miR-494-3p

hsa-miR-495-3p
hsa-miR-508-3p
hsa-miR-512-3p
hsa-miR-519c-5p
hsa-miR-519e-5p
hsa-miR-520d-5p
hsa-miR-520f-3p
hsa-miR-524-5p
hsa-miR-543
hsa-miR-552-3p
hsa-miR-579-3p
hsa-miR-588
hsa-miR-613
hsa-miR-616-3p
hsa-miR-708-5p
hsa-miR-874-3p
hsa-miR-9-5p
hsa-miR-942-5p
hsa-miR-96-5p
